# Effects on Immune Cells of a New 1,8-Naphthyridin-2-One Derivative and Its Analogues as Selective CB2 Agonists: Implications in Multiple Sclerosis

**DOI:** 10.1371/journal.pone.0062511

**Published:** 2013-05-01

**Authors:** Anna Maria Malfitano, Chiara Laezza, Alba D’Alessandro, Claudio Procaccini, Giuseppe Saccomanni, Tiziano Tuccinardi, Clementina Manera, Marco Macchia, Giuseppe Matarese, Patrizia Gazzerro, Maurizio Bifulco

**Affiliations:** 1 Dipartimento di Farmacia, Università di Salerno, Fisciano, Salerno, Italy; 2 Dipartimento di Medicina e Chirurgia, Università di Salern Facoltà di Medicina, Baronissi, Salerno, Italy; 3 Istituto di Endocrinologia e Oncologia Sperimentale, Consiglio Nazionale delle Ricerche (IEOS-CNR), c/o Dipartimento di Biologia e Patologia Cellulare e Molecolare, Università di Napoli “Federico II”, Napoli, Italy; 4 Laboratorio di Immunologia, Istituto di Endocrinologia e Oncologia Sperimentale, Consiglio Nazionale delle Ricerche (IEOS-CNR), c/o Dipartimento di Biologia e Patologia Cellulare e Molecolare, Università di Napoli “Federico II”, Napoli, Italy; 5 Dipartimento di Farmacia, Università of Pisa, Pisa, Italy; Institute Biomedical Research August Pi Sunyer (IDIBAPS) - Hospital Clinic of Barcelona, Spain

## Abstract

The efficacy of cannabinoids in the treatment of multiple sclerosis is widely documented; however their use is limited by psychoactivity mainly ascribed to the activation of the cannabinoid receptor CB1. Emerging findings support as alternative strategy in the treatment of neurodegenerative disorders, the application of compounds targeting the CB2 receptor, since likely unrelated to these side effects. Recently, a novel class of compounds, 1,8-naphthyridine, pyridine and quinoline derivatives have been demonstrated to show high CB2 receptor selectivity and affinity versus the CB1 receptor. Considering that the CB2 receptor is mainly expressed in cell and organs of the immune system, in this study we assessed the potential immune-modulatory effects of these compounds in activated lymphocytes isolated from MS patients with respect to healthy controls. These compounds blocked cell proliferation through a mechanism partially ascribed to the CB2 receptor, down-regulated TNF-α production and did not induce cell death. They also down-regulated Akt, Erk and NF-kB phosphorylation. Despite comparable effects observed in patients and healthy controls, these compounds, in particular, 1,8-naphthyridine and quinoline derivatives inhibited cell activation markers in MS patient derived lymphocytes more efficiently than in healthy control derived cells. Indeed, 1,8-naphthyridin-2-one derivative reduced the levels of Cox-2 in lymphocytes from patients whereas no effect was observed in control cells. Our findings suggest potential application of these drugs in neuro-inflammation, supporting further investigations of the effects of compounds in the therapy of MS, particularly on the aspects regarding activation and inflammation.

## Introduction

The endocannabinoid system (ECS) consists of receptors, transporters, endocannabinoids, and the enzymes involved in synthesis and degradation of endocannabinoids. Two major cannabinoid receptors, CB1 and CB2 have been described [1], [2]. CB1 is preferentially expressed in the central nervous system (CNS), [3], [4], [5] CB2 is predominantly expressed by immune cells [6], however evidence demonstrated its presence in the CNS [7], [8], [9]. Increasing reports suggest a role of the endocannabinoid system in a variety of physiological and pathophysiological conditions, including immunomodulation, [10] pain, cancer, [11], [12], [13], [14] psychiatric disorders [15] and immune-mediated diseases of the CNS such as multiple sclerosis (MS) [16]. In particular, MS results in focal areas of inflammation containing immune cell infiltrates and demyelination, [17] the prevailing view is that CD4+ T cells initiate the disease producing pro-inflammatory cytokines that drive the inflammatory process. In MS, these cells have specificity to ‘self antigens’ like myelin basic protein (MBP), myelin oligodendrocyte protein and proteolipid protein expressed by oligodendrocytes. Numerous studies reported the beneficial effects of cannabinoid derived-drugs in MS, [12], [16], [18] however adverse effects are mainly caused by the CB1 receptor [19]. This evidence supports as alternative strategy the use of CB2 agonists because of their minimal binding to CB1 receptors and therefore devoid of psychotropic effects. In macrophages/microglia, CB2 receptor stimulation suppressed the release of pro-inflammatory factors [20], [21] and some studies suggest the potential immunosuppressive role of CB2 selective ligands [22]. Recently, novel CB2 receptor agonists, 1,8-naphthyridine, pyridine and quinoline derivatives [23], [24], [25], [26], [27] have been designed with high CB2 affinity and CB2 versus CB1 selectivity in agreement with molecular modeling studies [23]. Some of these compounds exhibited pharmacological properties like inhibitory action on immunological human basophil activation mediated by the CB2 receptor [23], [24]. Based on these findings, showing high CB2 selectivity and inhibitory effect on immune cell activation, in this study we investigated the potential immune-modulatory and anti-inflammatory effects of the described 1,8-naphthyridine pyridine and quinoline derivatives in activated peripheral blood mononuclear cells (PBMC) isolated from both MS patients and healthy donors. We observed anti-proliferative effects on PBMC partially mediated by the CB2 receptor and associated with down-regulation of tumor necrosis factor (TNF)-α production. The inhibition of T cell activation markers was more pronounced in lymphocytes derived from MS patients than healthy donors. Furthermore, these drugs reduced the phosphorylation of Akt and the expression of cyclo-oxigenase-2 (COX-2). Our findings suggest potential application of these compounds as novel immune-suppressive and anti-inflammatory agents to potentially use in MS therapy.

## Methods

### Drugs

1,8-naphthyridin-2-one derivative: *N*-(4-methylcyclohexyl)-1-benzyl-1,8-naphthyridin-2(1*H*)-on-3-carboxamide (CB74), [24] quinolin-2-one derivative: *N*-cyclohepthyl-1-(2-morpholin-4-ylethyl)-quinolin-2(1*H*)-on-3-carboxamide (VL23); [27] pyridine-2-one derivative: *N*-cyclohepthyl-1-(p-fluorobenzyl)-1,2-dihydro-2-oxo-pyridine-3-carboxamide (AF4) [27]. Drugs and SR144528 were dissolved in DMSO. Vehicles were used as controls (not showed).

### Isolation of Human PBMC

All donors gave written informed consent in accordance with the Declaration of Helsinki to the use of their residual buffy coats for research purposes. All MS patients were recruited at the University Hospital “Federico II” of Naples and gave written informed consent. Approval of the local ethical committee of the University Hospital “Federico II” of Naples was obtained for this study. All patients recruited were females (matched for sex with controls), at diagnosis. All the patients were naïve to treatment and the expanded disability status scale (EDSS) was maximum 2. PBMC derived from buffy coats of healthy volunteers or from peripheral blood of MS patients were isolated as previously described [28]. Assays were performed in RPMI 1640 (Life Technologies, Paisley, UK) supplemented with penicillin/streptomycin (Life Technologies), 2 mM L-glutamine (Life Technologies) and 10% heat-inactivated FBS (Sigma Chemical Co., St Louis, MO, USA).

### Proliferation Assays on Human PBMC

PBMC isolated from ten MS patients and ten healthy donors (2×10^5^ cells per well) were cultured in triplicate in round-bottomed 96-well plates in a final volume of 200 µl of RPMI 10% FBS. Cells were stimulated with human MBP (10 µg/ml) (Sigma). Drugs were added to the cells to achieve final concentrations of 3 and 10 µM. In combinatory assays, cells isolated from healthy donors were treated with the CB2 antagonist SR144528 (0,5 µM) and the compounds were added at the concentration of 10 µM after SR144528. After 6 days of incubation, cells were pulsed with 1 µCi of ^3^H-thymidine (Amersham-Pharmacia Biotech, Cologno Monzese, Milano, Italy) and radioactivity was measured by scintillation counter (Wallac, Turku, Finland).

### TNF-α Production

Supernatants from cell cultures above described were harvested after 48 h to measure TNF-α production at drug concentration of 10 µM. Assays were performed by Human Fluorokine Multianalyte profiling (MAP) Base Kit (R&D system).

### Cell Vitality Assays

PBMC isolated from MS patients and healthy donors were cultured with MBP (10 µg/ml) in the presence and in the absence of the drugs at 10 µM in RPMI 10% FBS for 6 days in 24-well plates. After the incubation, cells were stained with trypan blue and counted by electron mycroscopy.

### Flow Cytometry Assays

PBMC (1×10^6^ cells) isolated from MS patients and healthy donors were cultured with MBP (10 µg/ml) and the drugs at 10 µM in RPMI 10% FBS for 6 days in 24-well plates. 10^5^ cells from each experimental condition were stained with the apoptotic marker Annexin V-FITC, or CD4-Cy-Chrome, CD54 (ICAM)-FITC and CD69-PE, (BD–Bioscience), incubated as previously described [18] and analyzed by flow cytometry. The analysis of the activation markers was performed gating on the lymphocyte region of CD4+T cells and the expression of the markers in drug treated cells was compared to that of activated and not treated cells. Flow cytometric analysis was performed by Summit v4.3. (Dako) program.

### Electrophoresis and Immunoblots

Cell extracts derived from MBP activated PBMC isolated from MS patients and healthy donors and treated with the compounds at 10 µM for 6 days were processed as previously described. [29] Primary antibodies (1∶1000) (host species: rabbit) specific for pAkt (Cell signalling), Akt (Cell signalling), Cox-2 (Santa Cruz Biotechnology Inc.), pNF-kB p65 (Cell signalling), NF-kB p65 (Cell signalling), pErk (Cell signalling), Erk (Cell signalling) were used. Immuno-detection of specific proteins was carried out with horseradish peroxidase-conjugated donkey anti-rabbit IgG (Bio-Rad, Life Science Research, Hercules, CA, USA), by enhanced chemiluminescence system (Amersham GE Healthcare). Actin (Santa Cruz Biotechnology Inc., anti-rabbit) was used as control.

### Statistical Analyses

The Mann-Whitney U-test was used for unrelated two-group analyses and the Kruskal-Wallis ANOVA test for three or more groups, using StatView software (Abacus Concepts Inc.). Results are expressed as mean ± SD. P values<0.05 were considered statistically significant.

## Results

### 1,8-naphthyridine, Pyridine and Quinoline Derivatives Inhibit PBMC Proliferation and TNF-α Production

**Figure 1:**
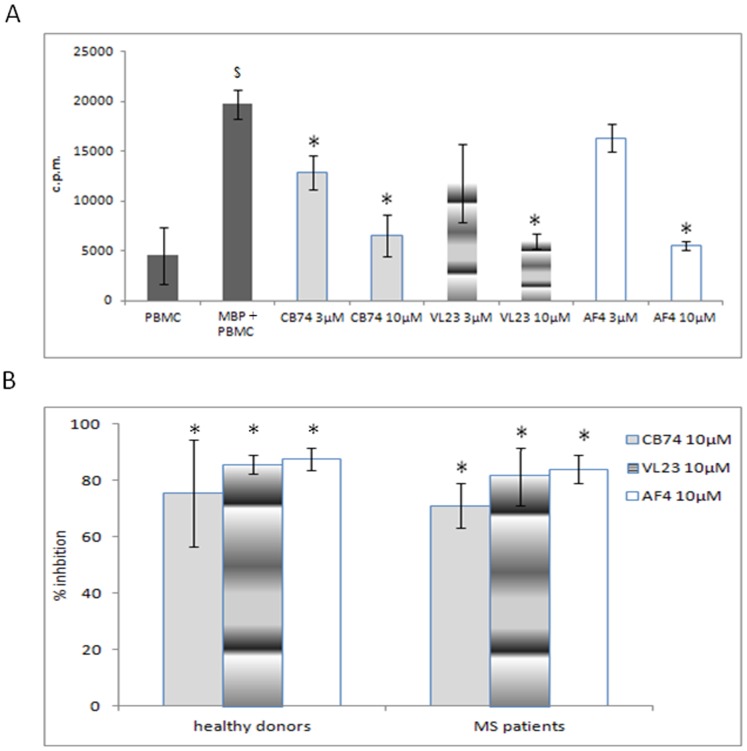
1,8-naphthyridine, pyridine and quinoline derivatives inhibit proliferation of MBP-activated PBMC. MBP activated PBMC of MS patients (2×10^5^ cells per well) were treated with the compounds at the indicated concentrations, in triplicate, for 6 days. Proliferation was measured after 1 h of ^3^H-thymidine incorporation (1 µCi). The counts per minutes (c.p.m.) ± the standard deviation of the triplicates of a representative experiment out of five are shown (A). The activation following MBP stimulation was evaluated with respect to MBP untreated cells, (PBMC in the figure) (^$^p<0,01). The statistical significance for each compound used at the concentrations of 3 and 10 µM, was calculated with respect to the MBP-activated cells (*p<0,05, MBP+PBMC in the figure). The histogram B represents the percent of inhibition of cell proliferation calculated with respect to drug untreated and MBP activated cells isolated from MS patients and healthy donors as indicated in the figure (*p<0,01). The percent of inhibition reported is the mean of five independent experiments and the bars represents the standard deviations.

**Figure 2:**
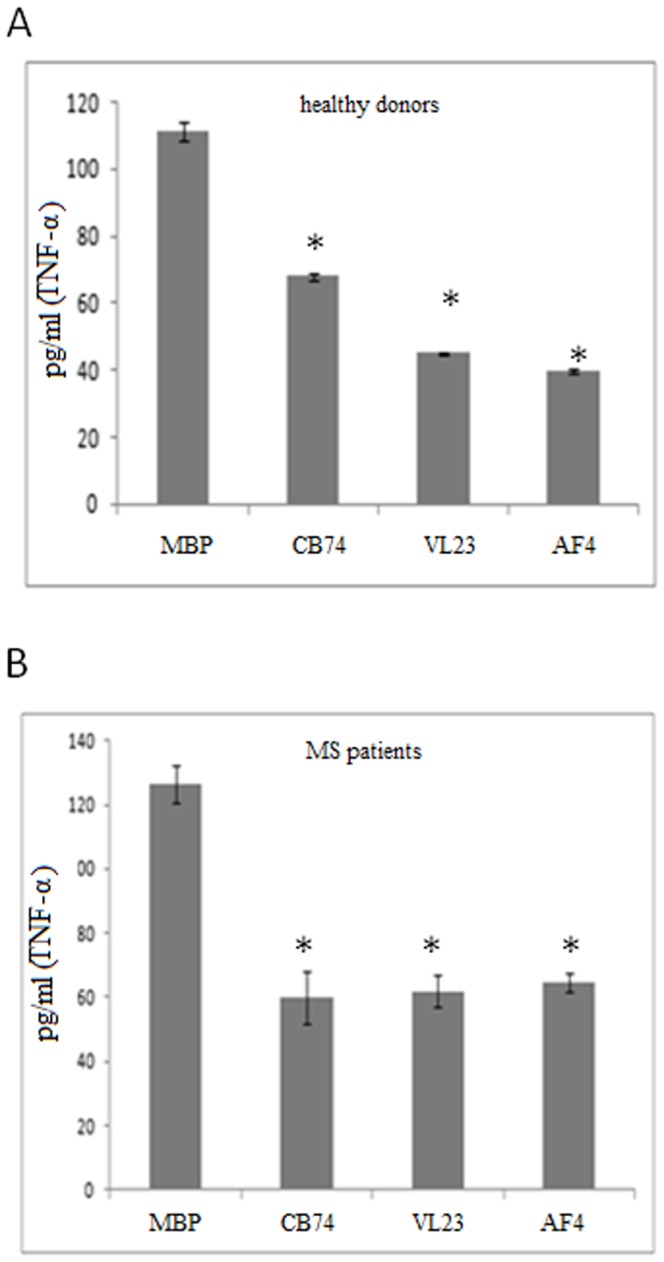
1,8-naphthyridine, pyridine and quinoline derivatives inhibit TNF-α production. The histograms represent the effects of CB74, VL23 and AF4 used at10 µM,on TNF-α concentration in supernatant collected after 48 h of culture of MBP activated PBMCs isolated from healthy controls (A) and patients (B) (*p<0,05 calculated with respect to MBP-activated PBMC, MBP in the figures). The histograms reported show a single experiment that is representative of four independent experiments with reproducible results.

We performed a kinetic of MBP treatment on PBMC derived from healthy donors at several time points (2 days, 4 days and 6 days) to find the best experimental condition of induction of proliferation (not showed). We observed that after 6 days of culture, MBP was able to induce an activation state in PBMC with respect to not treated cells in both MS patients (Fig. 1A) and control derived cells (not showed). Thus, the treatment with MBP at 6 days was considered to resemble the exposure to the myelin antigens of auto-reactive lymphocytes of MS patients. This condition was adopted in our experiments since considered useful to evidence potential effects of our compounds. The effect of our drugs was investigated in human PBMC isolated from MS patients and buffy coat of healthy donors. In MS patients, we observed a similar inhibition of the proliferative response of MBP activated PBMCs using CB74, VL23 and AF4 with respect to drug untreated cells (Fig. 1A). Similar effects on cell proliferation have been demonstrated on PBMCs derived from healthy donors (not showed). The percent of inhibition of PBMCs proliferation at the concentration of 10 µM (Fig. 1B) suggest that CB74, VL23 and AF4 exerted comparable effect on cell proliferation in both the cell from controls and patients (Fig. 1B). Although the use of micromolar doses might be considered too high to suggest a receptor mediated mechanism, we investigated whether a partial involvement of the CB2 receptor could be evoked. In order to establish the effects of the CB2 receptor antagonist SR144528, expected to enhance the number of proliferating cells when used in combination with our compounds, we used our drugs at 10 µM since higher anti-proliferative effect at this dose was observed. We found that the anti-proliferative effect of CB74, VL23 and AF4 was partially reverted by SR144528, *per se* inactive when used in the absence of our drugs. The effect of reversion of SR144528 was more pronounced with CB74, however a good extent of reversion was also observed with VL23 and AF4, as showed by the percent of cell recovery reported in table 1. Previous studies have also suggested that micromolar doses of synthetic CB2 ligands are effective in the modulation of immune cell function [10]. To assess if the anti-proliferative effects of our compounds could be associated to the down-regulation of inflammatory cytokine production, we measured the release of TNF-α in cell supernatants. We found that our compounds at 10 µM were able to significantly decrease MBP-induced TNF-α secretion by PBMCs, both in patients (Fig. 2A) and healthy donors (Fig. 2B).

**Table 1:** CB2 mediated effects of 1,8-naphthyridine, pyridine and quinoline derivatives.

Drugs	% inhibition	% recovery
	−SR144528	+SR144528
CB74	*75.4±19.0	^$^46.0±3.2
VL23	*85.9±3.2	^$^35.5±1.5
AF4	*87,8±3.9	^$^31.1±6.8

In table 1 we reported the percent of inhibition ± standard deviation of CB74, VL23 and AF4 at the concentration of 10 µM. The values are the mean of five independent experiments (these values correspond to the histogram reported in figure 1 B of healthy donors).The values reported in the presence of our drugs (without SR144528, -SR144528) are statistically significant with respect to MBP activated cells (*p<0,01).

The last colon of the table shows the percent of cell recovery ± standard deviation obtained culturing our compounds in the presence of SR144528 (+SR144528). The values reported are the mean of five independent experiments. The statistical analysis has been performed considering the values obtained with the drugs in the presence of SR144528 with respect to drug treated cells in the absence of SR144528 (^$^p<0,01).

### 1,8-naphthyridine, Pyridine and Quinoline Derivatives do not Affect Cell Viability

**Figure 3:**
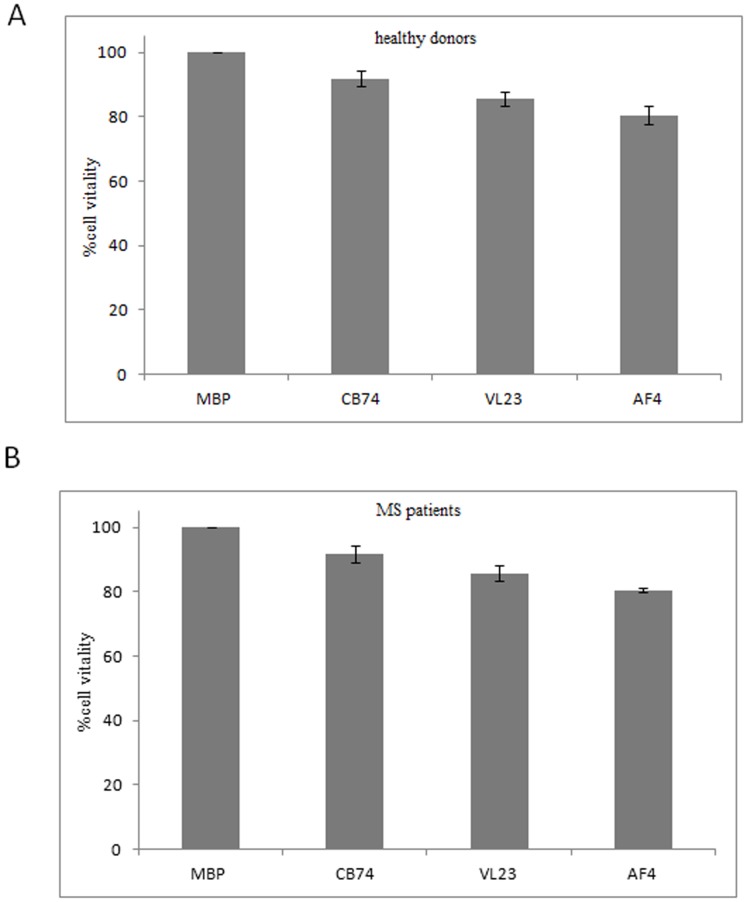
1,8-naphthyridine, pyridine and quinoline derivatives do not affect cell vitality. MBP activated PBMCs were treated with the drugs at the concentration of 10 µM and cultured for 6 days. After the incubation, cells were collected and stained with trypan blue. Cells were counted and the percent of cell vitality was evaluated on MBP activated PBMC in the presence and in the absence of our compounds. The histograms reported, show the percent of live cells in MBP activated PBMC (MBP in the figure) and in the presence of CB74, VL23 and AF4. PBMC were derived from healthy donors (A) and from MS patients (B).

In order to establish if the reported inhibition of proliferation and TNF-α release could be due to cell death rather than to the blocking of a specific cell signaling, we performed viability assays by trypan blue staining. We observed that at the dose of 10 µM, CB74, VL23 and AF4 did not affect viability of PBMCs derived from healthy donors (Fig. 3A) and patients (Fig. 3B) when compared to MBP activated cells and these findings were confirmed by Annexin V staining (data not showed).

### 1,8-naphthyridine, Pyridine and Quinoline Derivatives Inhibit T cell Activation Markers on MBP-activated PBMC

**Figure 4:**
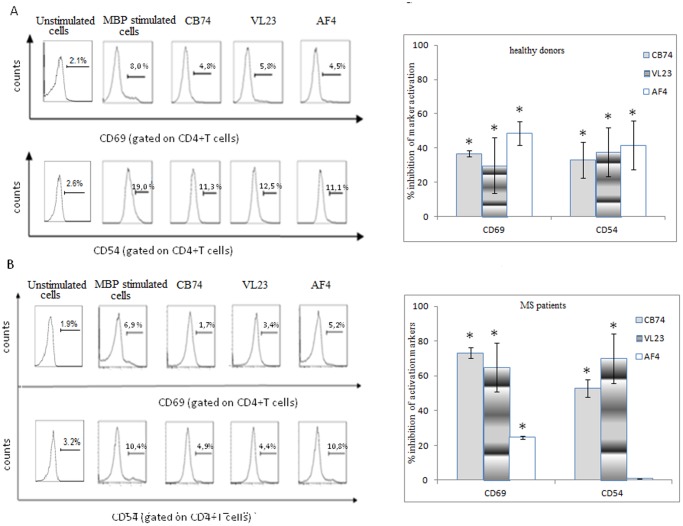
1,8-naphthyridine, pyridine and quinoline derivatives inhibit T cell activation markers, CD69 and CD54 adhesion molecule. MBP activated PBMC were treated with the drugs at the concentration of 10 µM and cultured for 6 days. After the incubation, cells were collected and stained with the antibodies indicated in the figure. Cells were analyzed by flow cytometry gating on the lymphocyte region of CD4+T cells. In the flow cytometric profile, representative of at least four independent experiments, cells expressing CD69 and CD54 in control PBMCs (Fig. 4A) and patient derived PBMCs (Fig. 4B), are showed along with the percent of expression of these markers. The control is represented by MBP activated cells. A further control, un-stimulated cells is also showed in the figure (A, B). The statistical analysis was performed on four independent experiments and the mean percent of marker inhibition ± the standard deviation of these experiments is reported on the right panels (*p<0,05 calculated with respect to MBP activated cells).

Furthermore, to assess whether the anti-proliferative effects of our compounds might be ascribed also to a down-regulation of cell activation, we examined the expression of some T cell activation markers, such as CD69 and the adhesion molecule CD54. In healthy subjects we observed that all the tested compounds inhibited CD69 and CD54 expression in a similar manner (Fig. 4A). In MS patients, CB74 and VL23 exerted a similar inhibition of the expression of CD69 and CD54, even better than that observed in control subjects, while AF4 was less efficient than the other compounds in inhibiting CD69 and had little effect on CD54 levels (Fig. 4B).

### 1,8-naphthyridine, Pyridine and Quinoline Derivatives affect Akt, NF-kB, Erk and Cox-2 Expression

**Figure 5:**
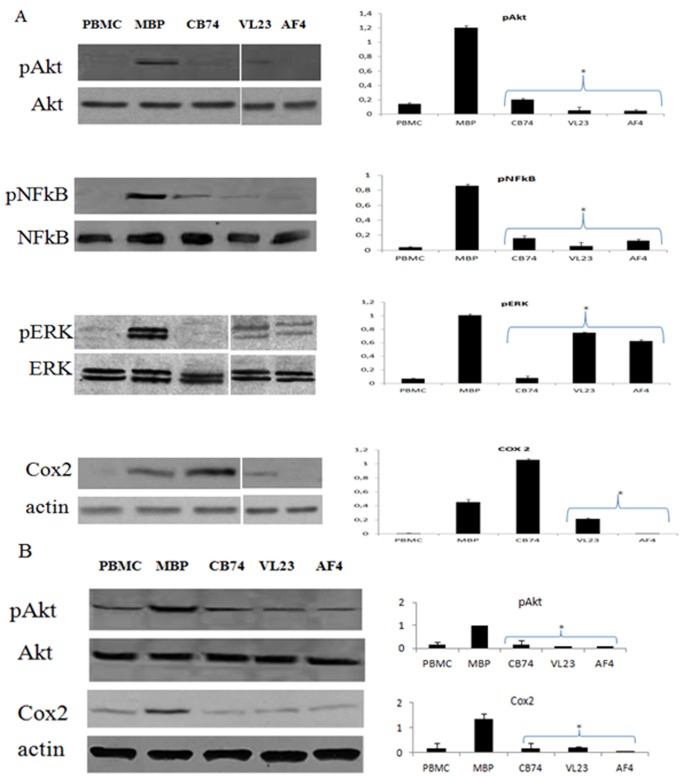
1,8-naphthyridine, pyridine and quinoline derivatives control Akt, NF-kB, Erk and Cox-2 expression. MBP activated PBMC were treated with the compounds at the concentration of 10 µM and cultured for 6 days. After the incubation, cell extracts were prepared and protein expression was determined by western blot analysis. In the blots, the bands of un-stimulated cells (PBMC in the figure), MBP activated cells (MBP in the figure) and the treatment with CB74, VL23 and AF4 are showed. The expression of phosphorylated Akt (pAkt) normalized on total Akt (Akt) and Cox-2 normalized on actin is showed in control cells (A) and in patient derived cells (B). The densitometric analysis is also reported in the histograms for both MS patient and control derived cells. In addition, in control cells (A), the protein expression of pNF-kB 65 kDa normalized on total NF-kB 65 kDa and pErk normalized on total Erk is also showed along with their relative densitometric analysis. The blots are representative of four independent experiments and the densitometric analysis reports the mean of the values of all the experiments ± the standard deviation (*p<0,01 significant inhibition calculated with respect to MBP activate PBMC).

It is widely reported that inflammatory mediators are involved in MS pathogenesis, in order to assess whether our compounds could modulate inflammatory pathways, we analyzed in MS patients and healthy donors, the expression of Akt, a relevant protein of the phosphatidylinositol 3′ –kinase (PI3K)-Akt signaling pathway. Akt can phosphorylate a number of proteins including IkB that frees NF-kB and allows it to translocate to the nucleus to bind and subsequently activate target genes. Among these targets, we analyzed the expression of the enzyme Cox-2 following treatment with our drugs. We observed that MBP treatment increased the phosphorylation of Akt with respect to un-stimulated cells (PBMC in the figure 5A, 5B) and our compounds down-regulated the expression of phosphorylated Akt in cells isolated from both controls (Fig. 5A) and patients (Fig. 5B). To better correlate our findings we also looked at the expression of NF-kB, a key factor of inflammation, to assess if Akt phosphorylation could be upstream of NF-kB activation. Furthermore, the signaling pathway of mitogen-activated protein kinase (MAPK) was also investigated to better define the targets of our drugs, in particular, we evaluated the expression of the extracellular signal-regulated kinases (Erk). We found that MBP activation induced the phosphorylation of NF-kB and Erk with respect to un-stimulated cells and the treatment with our compounds down-regulated their activation. The effect of CB74 on Erk was more pronounced with respect to VL23 and AF4 (Fig. 4A). MBP up-regulated also the levels of Cox-2 with respect to un-stimulated PBMC and drug treatment reduced its expression in cells isolated from controls except for CB74 (Fig. 5B). In patients, we observed similar effects and also CB74 inhibited Cox-2 levels (Fig. 5B).

## Discussion

Emerging studies support the high potential of the CB2 agonists in the treatment of neurodegenerative disorders including MS. In this study, we assessed the immune-modulatory effects of novel described CB2 selective agonists, 1,8-naphthyridine, pyridine and quinoline derivatives [23], [24], [27]. In particular, we compared the effects of these compounds in lymphocytes isolated from both MS patients and healthy donors. Our drugs efficiently blocked MBP activated PBMC proliferation (Fig. 1) and TNF-α production (Fig. 2) in both cell types. In order to exclude that such effects might be ascribed to cell death, we demonstrated that our compounds did not affect cell vitality (Fig. 3) and did not induce apoptosis (not showed), thus suggesting a block of a specific cell signaling. The down-regulation of TNF-α production is suggested to be desirable in MS since this cytokine is commonly enhanced during the course of MS. Our finding correlates with other studies showing that cannabinoids suppress TNF-α production [10]. Thus, since we observed similar effects on cell proliferation and TNF-α production in patients and controls, we used control cells to assess a potential mechanism of action of these compounds as higher cell number can be obtained from buffy coats of healthy donors. Since these drugs have high CB2 receptor affinity and selectivity, we explored if this receptor could be involved in the above described effects. Although the doses used might be considered high to suggest a receptor dependent mechanism of action, other studies have previously reported that concentrations in the micromolar range of synthetic cannabinoid agonists are often used to detect effects on immune cells [10]. Noteworthy, we observed that the anti-proliferative effect of our substances was partially mediated by the CB2 receptor since it was reverted by the CB2 receptor antagonist SR144528 (Table 1). In particular, the effect of SR144528 was more pronounced with CB74 that, among the compounds used in this study is the drug with higher CB2 receptor affinity, indeed for this compound a CB2 agonist activity has been previously determined [24]. The effect of VL23 and AF4 was also reverted but the reversion was lower compared to that obtained with CB74, these findings correlate with the affinity at the CB2 receptor reported by previous studies [27]. However, in prostate cancer cell lines, we recently showed a CB2 receptor mediated mechanism in the anti-proliferative effects of VL23 and AF4 [27]. To better characterize these effects, we explored their action on cell activation and adhesion. We found that on CD4+T cells, thought to initiate and drive the inflammatory process in MS, activation markers like CD69 and the adhesion molecule CD54 were potently down-regulated by CB74 and VL23. Of note, the compound with lower CB2 receptor affinity [27], AF4, did not affect cell activation, thus suggesting that higher CB2 affinity is needed to reduce activation in lymphocytes from patients. Previous studies also showed that the down-regulation of adhesion molecule by cannabinoid agonists interferes with the progression of MS [30], thus suggesting a therapeutic benefit derived by the use of modulators of these molecule involved in the regulation of transmigration of blood leukocytes across the blood–brain barrier in MS. The effects observed on CD69 and CD54 were higher in PBMC from patients than in control cells, thus supporting a likely specific effect of these compounds in lymphocytes from patients. A recent work showed that myelin activates FAK/Akt/NF-κB pathways and PI3K/Akt serves as upstream kinases for NF-κB activation by myelin [31]. In order to establish if our compounds could affect this signaling pathway, we analyzed the expression of Akt in MS patient and healthy donor derived cells. In MBP activated cells we found as expected, up-regulation of Akt phosphorylation while following drug treatment, we observed down-regulation of the phosphorylation of Akt. Similar effect was observed in control cells, thus suggesting that different effects of these compounds between lymphocytes from patients and healthy donors cannot be related to the modulation of this kinase.

To further explore the effects on this signaling pathway we also demonstrated that NF-kB expression is affected by our compounds, MBP activation induced NF-kB phosphorylation and our drugs blocked its phosphorylation in healthy donors (Fig. 5A). NF-kB is also a target of MAPK pathway, thus we examined the expression of Erk as component of this pathway. We observed that MBP activation induced Erk phosphorylation and our compounds down-regulated pErk (Fig. 5A) thus suggesting that these drugs are able to affect multiple signaling pathways. It is know that MS has a major inflammatory component that drives and orchestrates the disease. Our findings on NF-kB suggest that these compounds can also affect inflammation. In order to further assess potential anti-inflammatory properties of our compounds, we analyzed the expression of the enzyme Cox-2, target of NF-kB that is usually induced at site of inflammation and is a major player in inflammatory reaction in peripheral tissues. We found that CB74, VL23 and AF4 reduced Cox-2 levels in PBMC from patients; the effect was also maintained in control cells except for CB74. In healthy donors CB74 exerts a different action with respect to VL23 and AF4 at the same concentration. This different effect of CB74 in MS patients and in controls, evidences that CB74 can efficiently down-regulate Cox-2 expression only on auto-reactive lymphocytes or on lymphocytes frequently subjected to uncontrolled activation driven by autoimmune reaction. Thus, our finding suggests a specific anti-inflammatory effect of CB74 on patient derived cells since it is ineffective on control cells derived from healthy subjects. Overall, our results support potential applications of these compounds in inflammatory and autoimmune pathologies. Although immune-modulatory properties of CB2 agonists are known, this study shows for the first time peripheral effects in immune cells derived from MS patients of these CB2 selective compounds. Noteworthy, the effect of these drugs on cells derived from both MS patients and healthy donors are quite similar; however some effects seem specific in MS patients, like higher inhibition of cell activation in patients with respect to normal subjects and the effect on Cox-2 modulated by CB74 in cells of MS patients but not in controls. Our findings suggest that these molecules, partially acting by CB2 receptor mediated mechanism and likely unrelated to psychoactivity are good candidates for further studies aimed to establish their potential in the therapy of MS.
